# Additional effect of erenumab for patients with chronic migraine treated with onabotulinumtoxin A—real-world data from a preliminary cohort study

**DOI:** 10.3389/fneur.2024.1370503

**Published:** 2024-06-26

**Authors:** Tristan Koelsche, Petyo Nikolov, Valeria Koska, Jens Ingwersen, Robin Jansen, Ercan Arat, Sven G. Meuth, Philipp Albrecht, John-Ih Lee

**Affiliations:** ^1^Department of Neurology, Medical Faculty and University Hospital Düsseldorf, Heinrich Heine University Düsseldorf, Düsseldorf, Germany; ^2^Department of Neurology, Maria Hilf Clinics, Mönchengladbach, Germany

**Keywords:** migraine disability assessment, combination therapy of erenumab and onabotulinumtoxin A, prophylactic combination therapy, retrospective cohort study, quality of life in migraine

## Abstract

**Background:**

This preliminary retrospective cohort study investigates the potential additive prophylactic effect of erenumab, a fully human monoclonal antibody that blocks the calcitonin gene-related peptide receptor, in combination with ongoing onabotulinumtoxin A (onaBoNT-A) treatment in patients suffering from chronic migraine.

**Methods:**

The study included 218 patients and investigated the effects of adding erenumab to the existing treatment regimen. The primary outcome was the MIDAS (Migraine Disability Assessment) score assessed 3 months after the introduction of erenumab.

**Results:**

The results indicated a significant improvement of the MIDAS score, suggesting a reduction in migraine-related disability following the addition of erenumab to onaBoNT-A. In the inter group comparison, dual therapy showed a significantly greater reduction of the MIDAS when compared to a switch from onaBoNT-A to erenumab monotherapy, but not compared to initiation of onaBoNT-A monotherapy. It is hypothesized that the observed additive effects are due to the independent modes of action of erenumab and onabotulinumtoxin A.

**Conclusion:**

This study suggests that the combination of erenumab with onaBoNT-A may offer an improved approach for the treatment of chronic migraine in selected patients. However, the results highlight the need for prospective, controlled studies to validate these findings and determine the optimal combination of treatments tailored to the individual patient.

## Highlights

- Combination therapy with onabotulinumtoxin A and erenumab reduced MIDAS compared to prior onabotulinumtoxin A monotherapy.
- Addition of erenumab to onabotulinumtoxin A improved quality of life compared to onabotulinumtoxin A monotherapy.

## Introduction

Chronic migraine (CM) is characterized by headaches occurring on 15 or more days per month for more than 3 months, which, on at least 8 days per month, have the features of migraine (1). CM significantly impacts the quality of life, leading to substantial disability and reduced productivity and affects approximately 1%–2% of the population worldwide (2).

Current prophylactic treatments for CM include pharmacological and non-pharmacological approaches. Onabotulinumtoxin A (onaBoNT-A) has been widely used and has shown efficacy in reducing the frequency and severity of headaches (3). However, for a significant proportion of patients, treatment response to onaBoNT-A is not satisfactory, highlighting the need for additional therapeutic options.

In recent years, advances in the understanding of the pathophysiology of migraine have led to the development of novel therapeutic targets (4). One such target is the calcitonin gene-related peptide (CGRP), a neuropeptide implicated in the pathogenesis of migraine. Erenumab, a fully human monoclonal antibody (mAb) that blocks the CGRP receptor, is an approved treatment for migraine prevention (5). Clinical trials as well as real-world studies have demonstrated the safety and efficacy of erenumab in patients with episodic and CM (6–8). To our knowledge, no specific recommendations for the duration of a dual therapy with onaBoNT-A and erenumab have been published. However, consensus statements recommend a treatment pause for migraine preventive medication including erenumab or onaBoNT-A after 12 to 24 months in CM (9). Despite the efficacy of these treatments, some patients continue to experience significant disability.

The potential additive effect of erenumab in patients receiving onaBoNT-A treatment remains unclear. To address this gap in the literature we performed a retrospective analysis of real-world data from a cohort study to investigate the treatment response in patients who were initiated on erenumab while already receiving onaBoNT-A treatment for CM. We hypothesize that this combination therapy may lead to a significant improvement in the Migraine Disability Assessment (MIDAS) Score, a validated tool used to measure migraine-related disability (10).

## Methods

This study was a preliminary retrospective cohort study conducted on patients receiving onaBoNT-A at the outpatient clinic of Heinrich-Heine University Düsseldorf. Patients with CM receiving onaBoNT-A were included and divided into three groups. In the first group, onaBoNT-A monotherapy was initiated. The second group was switched from onaBoNT-A to erenumab monotherapy. The third was initiated on an additional treatment with erenumab. The study period spanned from January 2019 to March 2023. Eligible participants were adults aged 18–68 years with a diagnosis of CM as per the International Classification of Headache Disorders (ICHD-3) criteria. Patients in the switch and in the dual therapy groups had been on onaBoNT-A treatment for at least 6 months prior to the study commencement. Patients received 155–195 U of onaBoNT-A every 12 weeks, following the PREEMPT injection protocol (11) and follow the pain concept with a mean dose of 188 MU (SD ± 24) in the onaBoNT-A monotherapy group, 190 (SD ± 12) in the erenumab group prior to the treatment change and 192 MU (SD ± 8) in the dual therapy group. Patients not receiving onaBoNT-A, patients with other headache disorders and patients with contraindications for erenumab were excluded from the study.

Following the positive head-to-head study of Erenumab vs. Topiramate (12), the Federal Joint Committee in Germany issued the Amendment of the Pharmaceuticals Directive: Annex XII—Benefit Assessment of Medicinal Products with New Active Ingredients according to Section 35a SGB V Erenumab [Reassessment due to New Scientific Knowledge (Prophylaxis of Migraine)] (13). In this, they decided to facilitate reimbursement for erenumab due to a considerable additional benefit. Therefore, our cohort consists primarily of patients treated with erenumab.

Patients with no treatment response to onaBoNT-A after at least three cycles or no patient preference to discontinue onaBoNT-A were switched to erenumab monotherapy if no contraindications were present. Due to some but not satisfactory effect of their ongoing onaBoNT-A treatment (treatment response without achieving a sufficient reduction in headache days and improvement of quality of life as evaluated by the patient, but some subjective relief of pain and patient preference to continue treatment with onaBoNT-A) eligible participants were administered additional erenumab (70 mg) subcutaneously every 4 weeks. In the onaBoNT-A monotherapy group, 60 patients (36%), in the erenumab monotherapy group 12 (67%) and in the dual therapy group, 24 patients (68.6%) fulfilled the criteria of resistant migraine, while one patient (2.9%) had refractory migraine, according to the definition of the European headache federation consensus (14) 47 (29%) patients in the onaBoNT-A group, 6 (33%) of the erenumab monotherapy group and n9 (26%) patients in the dual therapy group were suffering from medication overuse headache (MOH) (25.7%) at the same time.

Data were collected retrospectively from medical records and questionnaires. Baseline data included demographic information, previous and current treatments, baseline MIDAS Score and the EQ-5D-5L Self-Complete Questionnaire. Follow-up data included MIDAS Score and the EQ-5D-5L Self-Complete Questionnaire at 3 months post-introduction of erenumab or after initiation of onaBoNT-A in the onaBoNT-A monotherapy group. Primary outcome measure was the change in MIDAS Score from baseline to 3 months post-introduction of erenumab or 3 months after initiation of onaBoNT-A in the onaBoNT-A monotherapy group. Also, we assessed monthly migraine days (MMD), monthly headache days (MHD), monthly analgesic drug use days (MDD) and mean headache intensity on the visual analog scale (VAS) during the last 3 months. Furthermore, we included the EQ-5D-5L Self-Complete Questionnaire to assess for quality-of-life changes.

The MIDAS questionnaire comprises 7 items, primarily focusing on the impact of migraines on daily activities. It assesses migraine-related disability over the past 3 months. Responses are based on the number of days a certain activity was limited due to migraines (ranging from 0 to more than 10 days). Scores are calculated by summing the number of days across items. The German MIDAS translation demonstrated intraclass correlation coefficients spanning from 0.884 to 0.994, with a value of 0.991 (95% CI: 0.982–0.995) for the MIDAS total score. Cronbach’s α for the entirety of the MIDAS was 0.69 during testing and 0.67 during retesting (15).

The EQ-5D-5L questionnaire consists of 5 items covering five dimensions of health: mobility, self-care, usual activities, pain/discomfort, and anxiety/depression. It measures health-related quality of life (HRQoL) across these dimensions. Each dimension has 5 response options ranging from “no problems” to “extreme problems.” The responses are converted into a health state index score using a predetermined value set, often country-specific, reflecting societal preferences for health states. This index score ranges from −0.59 to 1, where 1 represents full health, 0 represents death, and negative values represent states worse than death. EQ-5D-5L index values were computed, using the German (GER) Ludwig value set [Version 2.1 (Updated 08/04/2021)]. Descriptive statistics were used to summarize demographic and clinical characteristics. We tested for normal distribution using the Shapiro–Wilk test and the Kolmogorov–Smirnov test. Due to non-Gaussian distribution, Wilcoxon matched-pairs signed rank tests were used to compare the median MIDAS Score, as well as MMD, MHD, MDD, headache intensity, and the results of the EQ-5D-5L questionnaire before and after the introduction of erenumab. Then, we divided the dual therapy cohort into subgroups of patient’s with and without medication overuse headache (MOH) and resistant migraine and compared them using Chi Square Tests for inter subgroup differences and Wilcoxon matched-pairs signed rank tests for intra subgroup analysis before and after initiation of additive therapy. A *p*-value of less than 0.05 was considered statistically significant. For multiple testing, a Bonferroni correction was performed.

Comparisons of inter group differences between onaBoNT-A monotherapy, switch to erenumab monotherapy or dual therapy for MIDAS, EQ-5D-5L index score, MHD, MMD, MDD and headache intensity (VAS) were performed using the Kruskal-Wallis test and Dunn’s correction for multiple testing. All statistical analyses were performed using IBM SPSS statistics version 29.0.1.0 and GraphPad Prism Version 10.1.0.

The study was conducted in accordance with the Declaration of Helsinki and was approved by the local ethics committee (Study Number 5794R). All participants provided written consent to the study.

## Results

218 patients with CM were included in the study. Median age was 44 years (range 20–68 years). Most of the participants were female (96%). Participants were divided into three groups. The first was initiated on onaBoNT-A monotherapy, the second was switched to erenumab monotherapy and in the third group, erenumab was added to a preexisting onaBoNT-A treatment regime. All participants in the erenumab and dual therapy groups had been receiving onaBoNT-A treatment for at least 6 months prior to add-on erenumab therapy. Patients had been on 4 median preventive treatments before initiation of onaBoNT-A (range = 8; Table 1). We did not see either treatment discontinuation or severe adverse events in the observed cohort and period.

**Table 1:** Main baseline demographical and clinical characteristics.

Variable	OnaBoNT-A (*n* = 165)	Erenumab (*n* = 18)	Dual (*n* = 35)	Total (*n* = 218)
Female	151 (92)	18 (100)	33 (94)	209 (96)
Age	39 ± 18 (20–68)	53 ± 11 (25–68)	43 ± 13 (22–60)	44 ± 12 (20–68)
Latency onaBonT-A to erenumab days			617 ± 450 (170–2,176)	
Dose onaBoNT-A	188 ± 24 (155–195)	190 ± 12 (155–195)	192 ± 8 (155–195)	190 ± 14 (155–195)
MIDAS score	82 ± 76 (1–409)	80 ± 62 (7–206)	89 ± 66 (6–243)	83 ± 73 (1–409)
Resistant migraine	60 (36)	12 (67)	24 (68.6)	96 (44)
Refractory migraine	0 (0)	0 (0)	1 (2.9)	1 (1)
Medication overuse headache	47 (28.5)	6 (33)	9 (25.7)	62 (28)
MHD	16 ± 9 (2–30)	16 ± 9 (3–30)	16 ± 9 (3–31)	16 ± 9 (2–31)
MMD	12 ± 8 (0–30)	12 ± 7 (3–30)	14 ± 8 (2–31)	12 ± 8 (0–31)
MDD	12 ± 9 (0–31)	11 ± 6 (3–30)	11 ± 10 (1–31)	12 ± 9 (0–31)
Headache intensity (VAS)	7 ± 1 (4–10)	7 ± 1 (5–9)	7 ± 2 (4–10)	7 ± 1 (4–10)
Response rate (30% MHD reduction)	54 (33)	7 (39)	25 (71)	86 (39)
**Prior prophylactic medications**				
Propranolol	40 (24)	6 (33)	7 (20)	53 (24)
Bisoprolol	38 (23)	11 (61)	13 (37)	62 (28)
Metoprolol	65 (39)	9 (50)	18 (51)	92 (42)
Flunarizine	55 (33)	9 (50)	12 (34)	76 (35)
Topiramate	107 (65)	10 (56)	22 (63)	139 (64)
Amitriptyline	103 (62)	11 (61)	18 (51)	132 (61)
Valproate	18 (11)	5 (28)	3 (9)	26 (12)
Galcanezumab	1 (1)	1 (6)	1 (3)	3 (1)
Fremanezumab	2 (1)	1 (6)	1 (3)	4 (2)
Count of prior prophylactic medications	2 ± 2 (1–8)	4 ± 3 (2–8)	4 ± 2 (1–7)	3 ± 2 (1–8)
**Prophylactic medications during therapy**				
Propranolol	2 (1)	0 (0)	0 (0)	2 (1)
Bisoprolol	16 (10)	0 (0)	4 (11.4)	20 (9)
Metoprolol	22 (13)	2 (11)	3 (8.6)	27 (12)
Flunarizine	5 (3)	1 (6)	1 (2.9)	7 (3)
Topiramate	26 (16)	1 (1)	3 (8.6)	30 (14)
Amitriptyline	25 (15)	1 (1)	6 (17.1)	32 (15)
Valproate	2 (1)	0 (0)	0 (0)	2 (1)
Galcanezumab	0 (0)	0 (0)	0 (0)	0 (0)
Fremanezumab	0 (0)	0 (0)	0 (0)	0 (0)

Data are expressed as mean ± standard deviation (range) for scale variables or *n*, % of total for categorial variables. OnaBoNT-A, onabotulinumtoxin A; MIDAS, Migraine Disability Assessment; MHD, monthly headache days; MMD, monthly migraine days; MDD, monthly analgesic drug days; VAS, visual analog scale.

**Figure 1:**
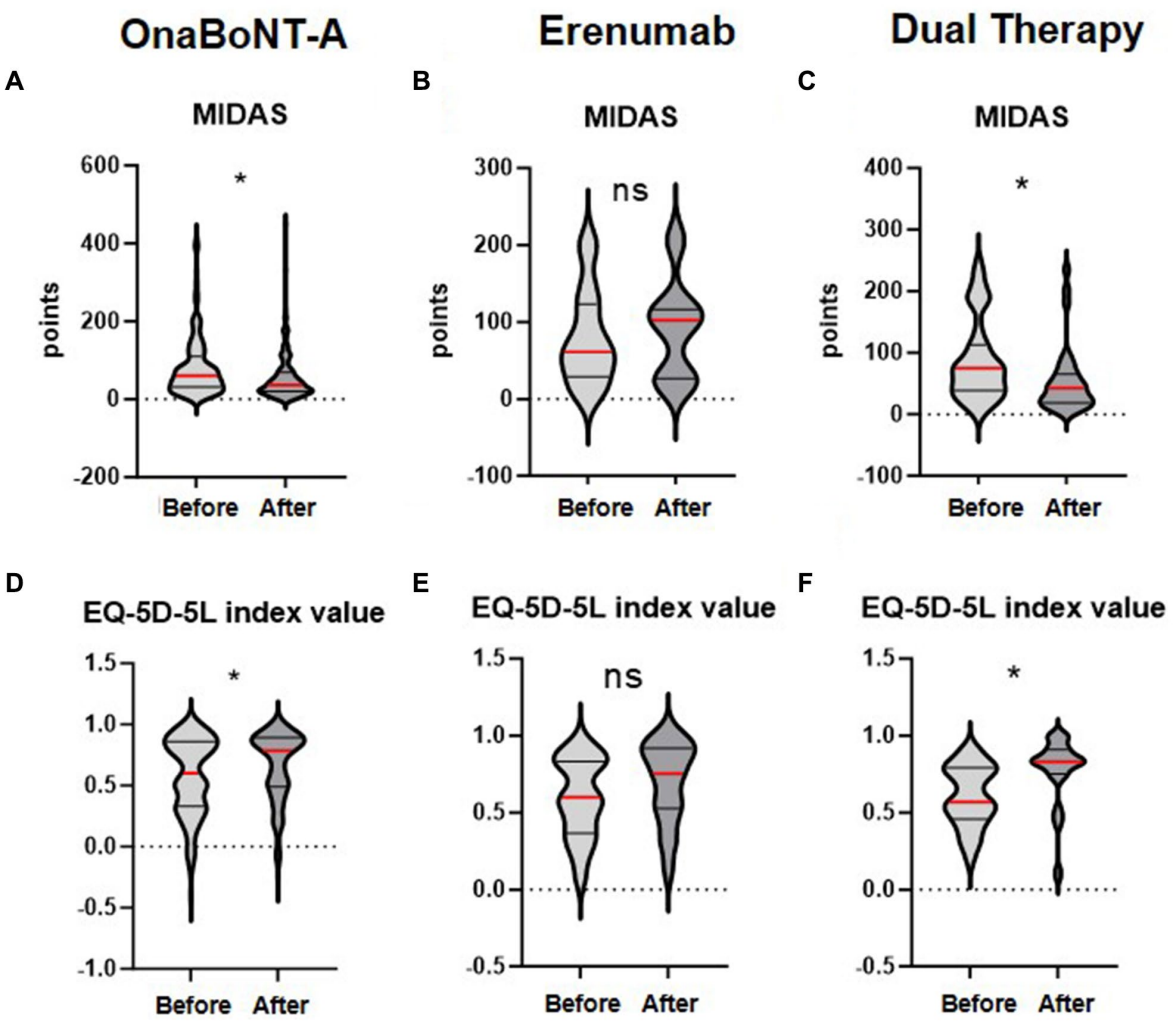
Changes in MIDAS and EQ-5D-5L index values before and after treatment with OnaBoNT-A monotherapy, Erenumab monotherapy, and dual therapy. The distribution of MIDAS scores **(A–C)** and EQ-5D-5L index values **(D–F)** before and after treatment with OnaBoNT-A monotherapy, erenumab monotherapy, and dual therapy (OnaBoNT-A plus erenumab). Panels **(A–C)** show the MIDAS values. **(A)** OnaBoNT-A monotherapy: Significant reduction in MIDAS scores after treatment. **(B)** Erenumab monotherapy: No significant change in MIDAS scores after treatment. **(C)** Dual therapy: Significant reduction in MIDAS scores after treatment. Panels **(D–F)** show the EQ-5D-5L index values. **(D)** OnaBoNT-A monotherapy: Significant improvement in EQ-5D-5L index values after treatment. **(E)** Erenumab monotherapy: No significant change in EQ-5D-5L index values after treatment. **(F)** Dual therapy: Significant improvement in EQ-5D-5L index values after treatment. Each violin plot shows the distribution of scores, with the width representing the density of data points and the central red line indicating the median value. Statistical analysis was performed using the Wilcoxon matched-pairs signed rank test. The asterisk (*) denotes statistical significance with Bonferroni adjusted *p* < 0.05, while “ns” indicates no significant difference.

Primary outcome measure was the change in MIDAS Score from baseline to 3 months post-introduction of erenumab or initiation of onaBoNT-A. At baseline, the mean MIDAS Scores were 82 (SD ± 76), 80 (SD ± 62), 89 (SD ± 66) in the onaBoNT-A monotherapy, the erenumab monotherapy and dual therapy group (Table 1). At the three-month follow-up, the mean MIDAS Scores were 56 (SD ± 61), 91 (SD ± 63) and 53 (SD ± 54) indicating a significant improvement in migraine-related disability in the onaBoNT-A monotherapy and dual therapy group (Table 2 for dual therapy, Supplementary Tables 1, 2 for onaBoNT-A and erenumab monotherapy; Figure 1; *p* < 0.0016). MHD improved from baseline mean 16 (SD ± 9) days per month in all three groups to 13 (SD ± 10, *p* < 0.0016) for onaBoNT-A, 14 (SD ± 10, *p* = 1) for erenumab monotherapy and 10 (SD ± 8, *p* = 0.0016) per month for dual therapy at 3-month follow-up. However, the reduction did not reach the Bonferroni adjusted significance level in the erenumab group (Supplementary Figure 1). We saw an improvement in MMD from a mean of 12 (SD ± 8) to 9 (SD ± 8, *p* = 0.0160) with onaBoNT-A monotherapy and from a mean of 14 (SD ± 8) to 8 days (SD ± 7, *p* = 0.0016) for combination therapy. A nonsignificant trend was found from a mean of 13 (SD ± 8) to 11 days (SD ± 8, *p* = 1) after switching to erenumab (Supplementary Figure 1). The reduction of monthly medication days from a mean of 12 (SD ± 9) to 11 (SD ± 8, *p* = 0.4672) days in the onaBoNT-A group, from 13 (SD ± 8) to 12 (SD ± 10, *p* = 1) in the erenumab monotherapy group and from 11 days (SD ± 10) to 7 days (SD ± 7, *p* = 0.0640) in the dual therapy group did not reach statistical significance after Bonferroni correction (Supplementary Figure 1). Headache intensity was non-significantly ameliorated from a mean of 7 (SD ± 2) points on the visual analog scale (VAS) to 6 points (SD ± 2, *p* = 0.0560) in the dual therapy group. In the onaBoNT-A group, mean headache intensity improved from 7.2 to 6.7 (SD ± 1 before ±2 after, *p* = 0.0256). In the erenumab monotherapy group, mean headache intensity stayed at 7 points (Supplementary Figure 1; SD ± 1 before and ± 2 after, *p* = 1).

**Table 2:** EQ-5D-5L and MIDAS score results.

	Before erenumab (*n* = 35)	After erenumab (*n* = 35)	Adjusted *p*-value
**EQ-5D-5L**			
Mobility	1.66 ± 0.94 (1–4)	1.49 ± 0.82 (1–4)	1
Self-care	1.20 ± 0.47 (1–3)	1.29 ± 0.71 (1–4)	1
Activity	2.46 ± 0.92 (1–4)	2.09 ± 0.85 (1–4)	1
Pain	3.46 ± 0.70 (2–5)	2.60 ± 1.01 (1–4)	**0.0016**
Anxiety	2.29 ± 0.93 (1–4)	1.94 ± 0.84 (1–4)	0.9872
EQ-5D-5l index value	0.61 ± 0.20 (0.19–0.91)	0.77 ± 0.22 (0.08–1)	**0.0016**
MIDAS score	89.06 ± 66.17 (6–243)	53.49 ± 53.63 (5–235)	**0.0016**
Missed days at work	11.89 ± 14.47 (0–72)	7.143 ± 10.49 (0–54)	**0.0016**
Performance at work < 50%	27.40 ± 23.80 (2–84)	13.37 ± 15.76 (0–60)	**0.0048**
Inability to do household work	19.49 ± 16.61 (2–72)	12.57 ± 13.97 (0–60)	0.0512
Performance household < 50%	17.80 ± 13.94 (1–50)	10.69 ± 11.94 (0–56)	**0.0144**
Days missed social events	16.66 ± 17.71 (0–80)	9.43 ± 12.52 (0–54)	**0.0304**
**MHD, MMD and medication days**			
Headache days (last 3 months)	44.20 ± 27.14 (3–92)	31.34 ± 25.14 (2–90)	**0.0032**
Headache intensity (VAS)	7.06 ± 1.68 (4–10)	6.26 ± 1.82 (3–9)	0.0560
MHD	16.20 ± 8.96 (3–31)	10.03 ± 7.68 (1–30)	**0.0016**
MMD	13.77 ± 8.20 (2–31)	8.11 ± 6.73 (1–26)	**0.0016**
MDD	11.37 ± 9.81 (1–31)	6.86 ± 7.16 (1–31)	0.0640

Values before and after addition of erenumab were compared via Wilcoxon matched-pairs signed rank test. Bonferroni correction for multiple testing (16 times) was performed and adjusted *p*-value < 0.05 was considered statistically significant. Data are expressed as mean ± standard deviation (range). OnaBoNT-A, onabotulinumtoxin A; MIDAS, Migraine Disability Assessment; MHD, monthly headache days; MMD, monthly migraine days; MDD, monthly analgesic drug days; VAS, visual analog scale. Bold values are statistically significant *p*-value <0.05 was considered statistically significant.

Regarding improvements in quality of life we found a significant decrease in the subscore for pain (*p* = 0.0064 onaBoNT-A, *p* = 0.0016 dual therapy), translating into a significant difference in the EQ-5D-5L index value (*p* = 0.0112 onaBoNT-A, p = 0.0016 dual therapy) in the subgroups with onaBoNT-A monotherapy and dual therapy, but not in the erenumab switch group (EQ-5D-5L pain: *p* = 1; Index: *p* = 1; Table 2; Supplementary Tables 1, 2; Figure 1).

In the dual therapy group, an analysis of differences between the subgroups of patients with (*n* = 9) and without (*n* = 26) prior medication overuse headache (MOH), as well as with (*n* = 24) and without resistant migraine (*n* = 11) before initiation of erenumab showed no significant differences (Supplementary Tables 3, 4).

When analyzing values of each subgroup distinctly before and after dual therapy, we gathered, that patients without MOH improved significantly in their self-reported days of reduced performance at work (*p* = 0.0119), in missed days at work (*p* = 0.0408), as well as the overall MIDAS Score (*p* = 0.0068), headache days during the prior 3 months (*p* = 0.0391), MHD (*p* = 0.17) and MMD (*p* = 0.0017), while patients with MOH did not (Supplementary Table 5).

Regarding the subgroups of patients suffering from resistant migraine (*n* = 24) compared to those without resistant migraine (*n* = 11), we found those with resistant migraine to have significantly decreased missed days at work (*p* < 0.0017) and MIDAS (*p* = 0.0306) after dual therapy. Additionally, they had decreased MHD (*p* < 0.0017), MMD (*p* = 0.0017) reduced headache intensity (*p* = 0.0017; Supplementary Table 6). In the dual therapy group, we compared the baseline values for MIDAS, EQ-5D-5L, MHD, MMD, and MDD with those assessed three months prior to the baseline visit. We did not find any significant differences (Supplementary Table 7).

**Figure 2:**
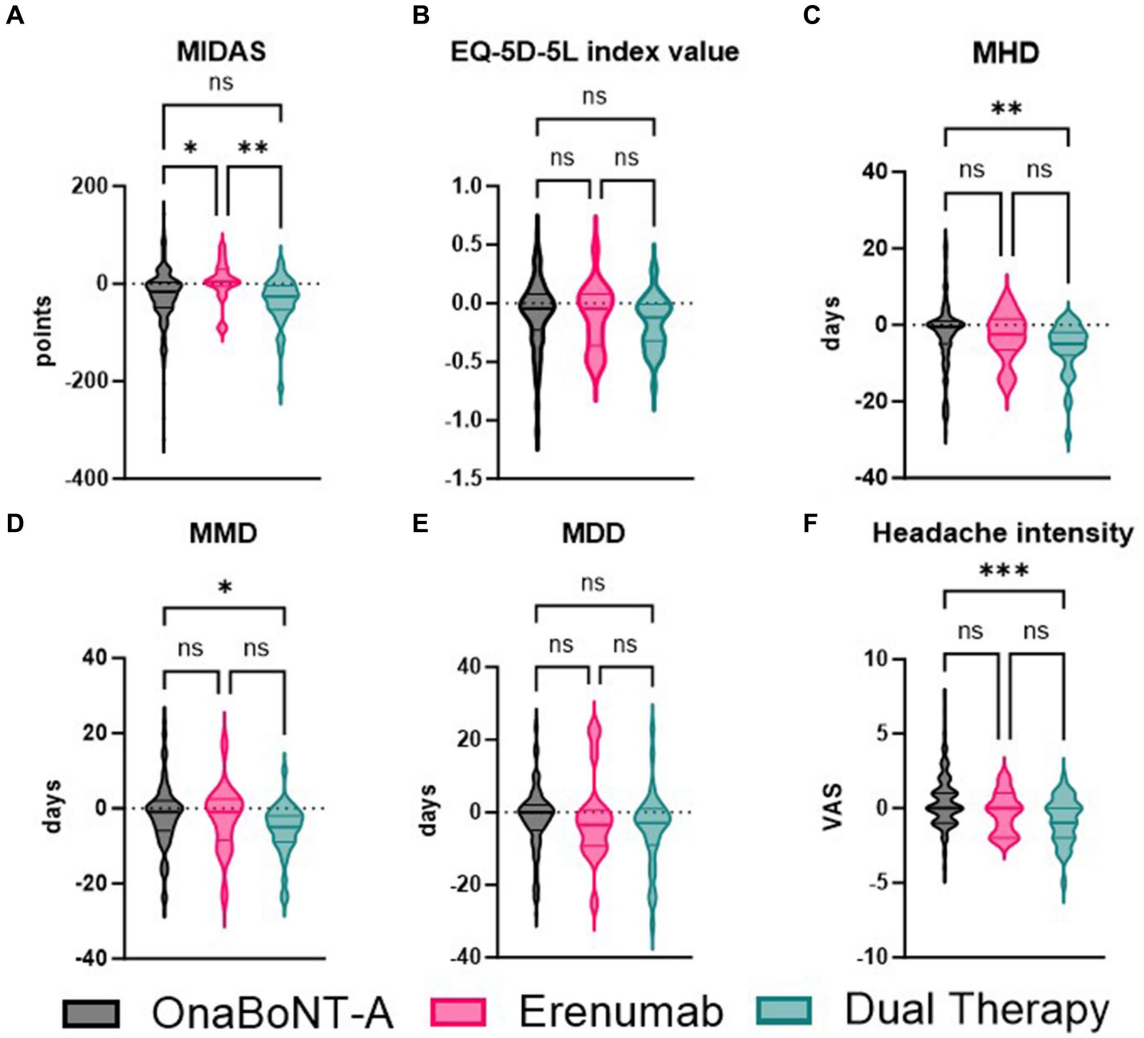
Comparative analysis of MIDAS, EQ-5D-5L index values, monthly headache days (MHD), monthly migraine days (MMD), monthly drug days (MDD), and headache intensity among OnaBoNT-A monotherapy, erenumab monotherapy, and dual therapy. This figure presents the violin plots comparing the distribution of various headache-related outcomes among patients treated with OnaBoNT-A monotherapy, erenumab monotherapy, and dual therapy (OnaBoNT-A plus erenumab). **(A)** MIDAS Scores: Significant reduction in MIDAS scores for OnaBoNT-A monotherapy and dual therapy compared to erenumab monotherapy. No significant difference between OnaBoNT-A and dual therapy. **(B)** EQ-5D-5L Index Values. No significant differences in EQ-5D-5L index values among the three treatment groups. **(C)** Monthly Headache Days (MHD). Significant reduction in MHD for dual therapy compared to OnaBoNT-A monotherapy. No significant differences between OnaBoNT-A and erenumab or erenumab and dual therapy. **(D)** Monthly Migraine Days (MMD). Significant reduction in MMD for dual therapy compared to OnaBoNT-A monotherapy. No significant differences between OnaBoNT-A and erenumab or erenumab and dual therapy. **(E)** monthly durg days (MDD). No significant differences in MDD among the three treatment groups. **(F)** Headache Intensity (VAS). Significant reduction in headache intensity for dual therapy compared to OnaBoNT-A monotherapy. No significant differences between OnaBoNT-A and erenumab or erenumab and dual therapy. Each violin plot shows the distribution of scores, with the width representing the density of data points and the central line indicating the median value. Statistical analysis was performed via Kruskal-Wallis tests with Dunn’s correction. The asterisks denote statistical significance with ^*^p < 0.05, **p < 0.01, and ***p < 0.001, while “ns” indicates no significant difference.

Furthermore, we compared the efficacy of onaBoNT-A monotherapy, switch to erenumab monotherapy, and dual therapy (onaBoNT-A plus erenumab) in reducing various headache-related outcomes, including MIDAS scores, EQ-5D-5L scores, monthly headache days, monthly migraine days, monthly medication days, and headache intensity (Figure 2; Table 3). The mean MIDAS score reduction for patients on onaBoNT-A monotherapy was by −26 (SD ± 60) points. For those switched to erenumab monotherapy, the mean MIDAS score increased by 4.071 (SD ± 49) points, while the dual therapy group had a mean reduction by −35 (SD ± 51) points. The Kruskal-Wallis test with Dunn’s correction showed a significant difference between onaBoNT-A and erenumab (*p* = 0.0473) and between erenumab and dual therapy (*p* = 0.0154), but no significant difference between onaBoNT-A and dual therapy (*p* = 0.7569).

**Table 3:** Reduction of MIDAS, MHD, MMD, and MDD, headache intensity of OnaBoNT-A monotherapy, switch to erenumab monotherapy or dual therapy after OnaBoNT-A monotherapy.

Variable	OnaBoNT-A (*n* = 165)	Erenumab (*n* = 18)	Dual therapy (*n* = 35)	*p*-value onaBoNT vs. erenumab	*p*-value onaBoNT vs. dual	*p*-value erenumab vs. dual
MIDAS Score	−26 ± 60 (−319–143)	4 ± 49 (−92–77)	−35 ± 51 (−214–45)	**0.0473**	0.7569	**0.0154**
EQ-5D-5L	−0.09 ± 0.29 (−1.1–0.6)	−0.11 ± 0.27 (−0.55–0.46)	−0.17 ± 0.23 (−0.71–0.30)	0.2779	0.2655	>0.9999
MHD	−2.55 ± 9.11 (−28–22)	−3.11 ± 6.46 (−16–7)	−6.17 ± 6.9 (−29–2)	>0.9999	**0.0077**	0.3235
MMD	−1.88 ± 8.58 (−25–23)	−2.47 ± 8.79 (−23–17)	−5.66 ± 6.85 (−24–10)	>0.9999	**0.0126**	0.2182
MDD	−1.6 ± 9.00 (−28–25)	−1.67 ± 11.95 (−25–23)	−4.51 ± 10.65 (−31–23)	0.7607	0.1671	>0.9999
Headache intensity (VAS)	0.45 ± 1.73 (−4–7)	−0.28 ± 1.36 (−2–2)	−0.80 ± 1.36 (−5–2)	0.3134	**0.0009**	>0.9999

Statistical comparison with Kruskal-Wallis test and Dunn’s correction. Adjusted *p*-value < 0.05 was considered statistically significant. Data are expressed as mean ± standard deviation (range). OnaBoNT-A, onabotulinumtoxin A; MIDAS, Migraine Disability Assessment; MHD, monthly headache days; MMD, monthly migraine days; MDD, monthly analgesic drug days; VAS, visual analog scale. Bold values are statistically significant *p*-value <0.05 was considered statistically significant.

The changes in EQ-5D-5L scores showed no significant differences among the groups. The onaBoNT-A group had a mean score increase of 0.09 (SD ± 0.29), the erenumab group had a mean score increase of 0.11 (SD ± 0.27), and the dual therapy group had a mean score increase of 0.17 (SD ± 0.23). The comparisons yielded *p*-values of 0.2779 for onaBoNT-A vs. erenumab, 0.2655 for onaBoNT-A vs. dual therapy, and >0.9999 for erenumab vs. dual therapy. For monthly headache days, the onaBoNT-A group showed a mean reduction of −2.55 days (SD ± 9.11). The erenumab group showed a slightly greater reduction of −3.11 days (SD ± 6.46), while the dual therapy group had the most substantial reduction of −6.17 days (SD ± 6.9). There was a significant difference between onaBoNT-A and dual therapy (*p* = 0.0077), but not between onaBoNT-A and erenumab (*p* > 0.9999) or erenumab and dual therapy (*p* = 0.3235). The reduction in monthly migraine days was −1.88 days (SD ± 8.58) for onaBoNT-A, −2.47 days (SD ± 8.79) for erenumab, and −5.66 days (SD ± 6.85) for dual therapy. Significant differences were found between onaBoNT-A and dual therapy (*p* = 0.0126), but not between onaBoNT-A and erenumab (*p* > 0.9999) or erenumab and dual therapy (*p* = 0.2182). The onaBoNT-A group had a mean reduction of −1.6 days (SD ± 9.00) in monthly medication days, while the erenumab group had a reduction of −1.67 (SD ± 11.95), and the dual therapy group had a reduction of −4.51 (SD ± 10.65). No significant differences were observed between onaBoNT-A and erenumab (*p* = 0.7607), onaBoNT-A and dual therapy (*p* = 0.1671), or erenumab and dual therapy (*p* > 0.9999). The mean change in headache intensity was 0.45 (SD ± 1.73) for onaBoNT-A, −0.28 (SD ± 1.36) for erenumab, and −0.80 (SD ± 1.36) for dual therapy. A significant difference was observed between onaBoNT-A and dual therapy (*p* = 0.0009), but not between onaBoNT-A and erenumab (*p* = 0.3134) or erenumab and dual therapy (*p* > 0.9999).

## Discussion

Our findings suggest that erenumab can be a valuable addition to the treatment regimen for CM patients already receiving onaBoNT-A. The additional improvement in MIDAS Score indicates a reduction in migraine-related disability, leading to improved quality of life for these patients as shown by the amelioration of the EQ-5D-5L index value.

While onaBoNT-A demonstrated good efficacy in a part of our patients, in a subgroup, therapeutic outcomes did not achieve a satisfactory symptom relief. Consequently, depending on the extent of some subjective improvement through onaBoNT-A and patient preference, we decided to switch the regimen to erenumab monotherapy or treat with erenumab additionally. The clinical rationale was driven by our commitment to enhance patient care and improve therapeutic outcomes, particularly when initial treatments may not yield optimal results. Our retrospective study demonstrated a significant improvement in MIDAS scores for CM patients receiving erenumab as an adjunct to onaBoNT-A treatment. Additionally, we report an improvement of quality of life for patients with CM. Due to the non-randomized, retrospective design of our study and therefore risk of selection bias, as well as due to the small sample size of the groups, comparability of dual therapy to erenumab or onaBoNT-A monotherapy is limited.

Even though we are aware of the limited validity due to the very small sample sizes, we conducted further analyses on subgroups of our cohort regarding concomitant medication overuse headache and treatment resistant migraine. We found no significant effectiveness of combining onaBoNT-A and erenumab in patients with MOH. Patients with per definition resistant migraine profited in our study from dual therapy, as suggested recently (16).

The results of the inter group comparisons demonstrate the varying efficacies of onaBoNT-A monotherapy, erenumab monotherapy, and dual therapy (onaBoNT-A plus erenumab) in treating chronic migraines. The data indicate that dual therapy often provides more substantial benefits across several key metrics compared to monotherapy with either onaBoNT-A or erenumab alone.

Dual therapy led to a greater improvement in the pain/discomfort and the usual activities subscore of the EQ-5D-5L questionnaire when compared to onaBoNT-A monotherapy. In comparison to the group that was switched to erenumab we found a significantly greater improvement in the pain/discomfort subscore and the MIDAS. Furthermore, dual therapy did not perform worse than monotherapy in any of the observed metrics. However, the intergroup comparisons come with several limitations. Firstly, the sample size for the erenumab group was relatively small (n = 18), which may affect the generalizability of the results. Additionally, the retrospective nature of the study and the lack of randomization may introduce bias.

Our findings suggest a potential synergistic effect of erenumab and onaBoNT-A. It has been proposed that erenumab’s inhibition of the CGRP receptor could modulate nociceptive transmission and central sensitization, while onaBoNT-A’s inhibition of neurotransmitter release could reduce peripheral sensitization (17, 18). In a recent review, the inhibition of Aδ-fiber activation through fremanezumab and the modulation of predominately C-fibers through onaBoNT-A have been discussed as synergistic mechanisms (19).

Our study’s retrospective design, the dominance of female patients and the single-center setting may limit the generalizability of our findings. Possible explanations for the female predominance are the higher prevalence of chronic migraine in women generally and the higher acceptance and knowledge of, as well as familiarity with botulinum toxin treatments, also for cosmetic procedures, in women (20). Additionally, the lack of a control group and the potential for confounding factors, such as concurrent medications and lifestyle changes, may have influenced the results.

Few studies have delivered conflicting results on the efficacy of a combination therapy of onaBoNT-A and erenumab. Jaimes et al. found in their cohort, that dual therapy with erenumab and onaBoNT-A was less effective than erenumab alone in reducing MHD and in raising the percentage of improvement in CM patients (21). Mechtler et al. described in their real-world study a beneficiary effect in monthly headache days of the addition of either erenumab (56.7%), fremanezumab (42.6%) or galcanezumab (0.7%) to patients already receiving onaBoNT-A (22). Positive results were further obtained in CM patients without aura (23) and in another retrospective chart review by Blumenfeld et al. (24). Further data from a real-life cohort study, supporting a synergistic mechanism have been published by Nandyala et al. (25). However, none of the aforementioned studies included further quality of life measurements.

A recent systematic review and meta-analysis has provided promising results for the combined treatment of onaBoNT-A and anti-CGRP mAbs in CM management. The combined therapy demonstrated a significant reduction in MHD for up to 58.8% of patients. In comparison, anti-CGRP Abs alone reduced MHD by 1.94 days from baseline, and onaBoNT-A alone by 1.86 days. The study revealed that the combination therapy resulted in a greater reduction of 2.67 MHD compared to onaBoNT-A alone, providing moderate certainty of evidence (26). Most evidence exists for the mAb erenumab, while only one study, conducted by Toni and colleagues reported on a relatively higher number of patients receiving fremanezumab (27).

Future research should investigate the long-term efficacy and safety of combined erenumab and onaBoNT-A treatment in CM patients, as well as potential predictive factors for treatment response. Especially patients experiencing a wearing off phenomenon of onaBoNT-A alone could profit from an additive anti-CGRP mAb (28). Randomized controlled trials are warranted to establish the comparative effectiveness of erenumab and onaBoNT-A alone or in combination, and to elucidate the underlying mechanisms of their synergistic effect. It would also be valuable to explore whether the observed additive effect extends to other monoclonal antibodies targeting the CGRP pathway, especially galcanezumab and eptinezumab.

## Conclusion

The addition of erenumab to onaBoNT-A treatment in CM patients was associated with a significant improvement in MIDAS scores, indicating a potential additive effect. These findings may have implications for the management of CM patients with persisting symptoms, although further research is required to confirm these results and elucidate the underlying mechanisms.

## Data availability statement

The raw data supporting the conclusions of this article will be made available by the authors, without undue reservation.

## Ethics statement

The studies involving humans were approved by Ethikkommission an der Med. Fakultät der HHU Düsseldorf. The studies were conducted in accordance with the local legislation and institutional requirements. The participants provided their written informed consent to participate in this study.

## Author contributions

TK: Writing – review & editing, Writing – original draft, Visualization, Methodology, Investigation, Formal analysis, Data curation, Conceptualization. PN: Writing – review & editing, Investigation. VK: Writing – review & editing, Investigation. JI: Writing – review & editing. RJ: Writing – review & editing, Investigation. EA: Writing – review & editing. SM: Writing – review & editing. PA: Writing – review & editing, Validation, Supervision, Project administration, Conceptualization. J-IL: Writing – review & editing, Validation, Supervision, Resources, Project administration, Methodology, Data curation, Conceptualization.
