# Supplementary material for: Additional effect of erenumab for patients with chronic migraine treated with onabotulinumtoxin A—real-world data from a preliminary cohort study
Source: Front Neurol. 2024 Jun 26;15:1370503. doi: 10.3389/fneur.2024.1370503 (PMC11234259; doi:10.3389/fneur.2024.1370503)
Supplement: Supplementary file 1 [file Data_Sheet_1.docx]

**Supplemental Tables**

**Supplemental Table 1:** **EQ-5D-5L and MIDAS Score results of the OnaBoNT-A monotherapy group.** Values before and after initiation of OnaBoNT-A were compared via Wilcoxon matched-pairs signed rank test. Bonferroni correction for multiple testing was performed and adjusted p-value < 0.05 was considered statistically significant.

|  | **Before onaBoNT-A** | **After onaBoNT-A** | **Adjusted p-value** |
| --- | --- | --- | --- |
| **EQ-5D-5L** |  |  |  |
| Mobility | 1.85 ± 1.11 (1-5) | 1.69 ± 0.96 (1-5) | 0.8240 |
| Selfcare | 1.40 ± 0.85 (1-4) | 1.30 ± 0.68 (1-4) | 1 |
| Activity | 2.42 ± 1.15 (1-5) | 2.15 ± 1.07 (1-5) | **0.0336** |
| Pain | 3.33 ± 1.03 (1-5) | 3.01 ± 1.04 (1-5) | **0.0064** |
| Anxiety | 2.24 ± 1.10 (1-5) | 2.18 ± 1.13 (1-5) | 1 |
| EQ-5D-5L index value | 0.58 ± 0.31 (-0.4-1) | 0.67 ± 0.28 (-0.26-1) | **0.0112** |
| **MIDAS Score** | 81.51 ± 76.05 (1-409) | 53.49 ± 53.63 (5-235) | **0.0016** |
| Missed days at work | 12.21 ± 18.29 (0-90) | 8.21 ± 14.36 (0-90) | 0.0592 |
| Performance at work < 50% | 18.85 ± 20.51 (0-91) | 12.71 ± 16.54 (0-91) | **0.0016** |
| Inability to do household work | 16.78 ± 17.02 (0-90) | 12.55 ± 15.13 (0-90) | **0.0016** |
| Performance household < 50% | 17.04 ± 17.26 (0-90) | 12.69 ± 15.74 (0-90) | **0.0016** |
| Days missed social events | 16.62 ± 20.07 (0-90) | 9.52 ± 12.26 (0-90) | **0.0016** |
| **MHD, MMD and medication days** |  |  |  |
| Headache days (last 3 months) | 37.49 ± 27.70 (0-90) | 32.87 ± 32.87 (0-92) | 0.1872 |
| Headache intensity (VAS) | 7.16 ± 1.37 (4-10) | 6.71 ± 1.77 (0-10) | **0.0256** |
| MHD | 15.71 ± 9.30 (2-30) | 13.16 ± 9.82 (0-31) | **0.0016** |
| MMD | 11.63 ± 8.10 (0-30) | 9.74 ± 7.89 (0-30) | **0.0016** |
| MDD | 12.29 ± 8.75 (0-31) | 10.68 ± 8.01 (0-31) | 0.4672 |

*Data are expressed as mean ± standard deviation (range).*

*onaBoNT-A* onabotulinumtoxin A, *MIDAS* Migraine Disability Assessment, *MHD* monthly headache days, *MMD* monthly migraine days, *MDD* monthly analgesic drug days, *VAS* visual analog scale.

**Supplemental Table 2:** **EQ-5D-5L and MIDAS Score results of the erenumab monotherapy switch group.** Values before and after switch to erenumab were compared via Wilcoxon matched-pairs signed rank test. Bonferroni correction for multiple testing was performed and adjusted p-value < 0.05 was considered statistically significant.

|  | **onaBoNT-A monotherapy** | **After switch from onaBoNT-A to erenumab monotherapy** | **Adjusted p-value** |
| --- | --- | --- | --- |
| **EQ-5D-5L** |  |  |  |
| Mobility | 1.61 ± 0.98 (1-4) | 1.44 ± 0.78 (1-3) | 1 |
| Selfcare | 1.40 ± 0.85 (1-4) | 1.28 ± 0.67 (1-3) | 1 |
| Activity | 2.17 ± 1.20 (1-4) | 2.28 ± 1.13 (1-4) | 1 |
| Pain | 3.39 ± 0.78 (2-4) | 2.89 ± 1.02 (1-4) | 1 |
| Anxiety | 2.39 ± 1.04 (1-4) | 2.00 ± 1.24 (1-4) | 1 |
| EQ-5D-5L index value | 0.59 ± 0.26 (0.08-0.94) | 0.70 ± 0.26 (0.13-1) | 1 |
| **MIDAS Score** | 80.31 ± 62.11 (7-206) | 91.43 ± 63.13 (16-210) | 1 |
| Missed days at work | 15.19 ± 20.25 (0-70) | 13.13 ± 14.14 (0-54) | 1 |
| Performance at work < 50% | 17.72 ± 17.00 (0-50) | 21.50 ± 22.05 (5-90) | 1 |
| Inability to do household work | 14.59 ± 15.67 (0-60) | 15.88 ± 13.68 (0-54) | 1 |
| Performance household < 50% | 15.65 ± 13.33 (0-50) | 16.94 ± 16.64 (0-60) | 1 |
| Days missed social events | 18.12 ± 17.62 (0-60) | 16.29 ± 15.87 (0-54) | 1 |
| **MHD, MMD and MDD** |  |  |  |
| Headache days (last 3 months) | 46.11 ± 39.72 (0-92) | 39.72 ± 34.40 (2-92) | 1 |
| Headache intensity (VAS) | 6.83 ± 1.38 (5-9) | 6.56 ± 1.79 (3-9) | 1 |
| MHD | 17.06 ± 9.10 (3-30) | 13.94 ± 10.21 (2-30) | 1 |
| MMD | 13.11 ± 8.26 (3-30) | 11.24 ± 8.17 (2-31) | 1 |
| MDD | 13.17 ± 8.18 (3-30) | 11.50 ± 9.64 (2-31) | 1 |

*Data are expressed as mean ± standard deviation (range).*

*onaBoNT-A* onabotulinumtoxin A, *MIDAS* Migraine Disability Assessment, *MHD* monthly headache days, *MMD* monthly migraine days, *MDD* monthly analgesic drug days, *VAS* visual analog scale.

**Supplemental Table 3:** **Medication overuse headache (MOH) and outcome parameters before and after Erenumab.** Patients with and without MOH were compared via Chi-Square Test. Bonferroni correction for multiple testing was performed and adjusted p-value < 0.05 was deemed statistically significant.

|  |  | **No MOH (n=26)** | | | **MOH (n=9)** | | | **Adjusted p-value** |
| --- | --- | --- | --- | --- | --- | --- | --- | --- |
| **Variables** |  | **Mean** | **SD** | **range** | **Mean** | **SD** | **range** |  |
| Mobility | before | 2 | 1 | 1-4 | 2 | 1 | 1-3 | 1 |
|  | after | 2 | 1 | 1-4 | 1 | 1 | 1-3 | 1 |
| Selfcare | before | 1 | 0 | 1-3 | 1 | 1 | 1-2 | 1 |
|  | after | 1 | 1 | 1-4 | 1 | 0 | 1-1 | 1 |
| Activity | before | 2 | 1 | 1-4 | 3 | 1 | 1-4 | 1 |
|  | after | 2 | 1 | 1-4 | 2 | 1 | 1-3 | 1 |
| Pain | before | 3 | 1 | 2-5 | 3 | 1 | 3-4 | 1 |
|  | after | 3 | 1 | 1-4 | 2 | 1 | 1-3 | 1 |
| Anxiety | before | 2 | 1 | 1-4 | 3 | 1 | 1-4 | 1 |
|  | after | 2 | 1 | 1-4 | 2 | 1 | 1-3 | 1 |
| EQ-5D-5L Index | before | 0.61 | 0.20 | 0.19-0.91 | 0.59 | 0.18 | 0.29-0.8 | 1 |
|  | after | 0.75 | 0.25 | 0.08-1.0 | 0.85 | 0.09 | 0.72-1.0 | 1 |
| Missed days at work | before | 11 | 16 | 0-72 | 14 | 9 | 3-33 | 1 |
|  | after | 6 | 11 | 0-54 | 10 | 8 | 3-26 | 1 |
| Performance at work < 50% | before | 22 | 21 | 0-80 | 28 | 21 | 7-62 | 1 |
|  | after | 14 | 16 | 0-58 | 14 | 18 | 1-60 | 1 |
| Inability to do household work | before | 16 | 13 | 2-50 | 29 | 23 | 3-72 | 1 |
|  | after | 11 | 12 | 0-54 | 16 | 19 | 1-60 | 1 |
| Household < 50% | before | 16 | 15 | 1-50 | 23 | 11 | 11-40 | 1 |
|  | after | 9 | 11 | 0-56 | 15 | 14 | 2-46 | 1 |
| Days missed social events | before | 15 | 18 | 0-80 | 22 | 18 | 5-46 | 1 |
|  | after | 9 | 11 | 0-54 | 11 | 16 | 0-50 | 1 |
| MIDAS Score | before | 79 | 63 | 6-243 | 117 | 72 | 39-224 | 1 |
|  | after | 49 | 48 | 5-202 | 65 | 69 | 10-235 | 1 |
| Headache days (last 3 months) | before | 40 | 27 | 3-92 | 55 | 26 | 30-92 | 1 |
|  | after | 30 | 25 | 2-90 | 37 | 27 | 7-80 | 1 |
| Headache intensity | before | 7 | 2 | 4-10 | 7 | 2 | 4-9 | 1 |
|  | after | 6 | 2 | 3-9 | 6 | 2 | 3-9 | 1 |
| MHD | before | 15 | 9 | 3-31 | 20 | 9 | 10-31 | 1 |
|  | after | 10 | 8 | 2-30 | 11 | 8 | 1-27 | 1 |
| MMD | before | 13 | 8 | 2-31 | 17 | 8 | 5-31 | 1 |
|  | after | 8 | 7 | 1-26 | 8 | 5 | 1-17 | 1 |
| MDD | before | 6 | 3 | 1-14 | 27 | 6 | 18-31 | 0.204 |
|  | after | 6 | 6 | 1-27 | 9 | 9 | 0-31 | 1 |

*MOH* medication overuse headache, *MIDAS* Migraine Disability Assessment, *MHD* monthly headache days, *MMD* monthly migraine days, *MDD* monthly analgesic drug days.

**Supplemental Table 4: Resistant migraine and outcome parameters before and after Erenumab.** Patients with and without resistant migraine were compared via Chi-Square Test. Bonferroni correction for multiple testing was performed and adjusted p-value < 0.05 was deemed statistically significant.

|  |  | **No Resistant migraine (n=11)** | | | **Resistant migraine (n=24)** | | | **Adjusted p-value** |
| --- | --- | --- | --- | --- | --- | --- | --- | --- |
| **Variables** |  | **Mean** | **SD** | **range** | **Mean** | **SD** | **range** |  |
| Mobility | before | 2 | 1 | 1-4 | 2 | 1 | 1-4 | 1 |
|  | after | 1 | 1 | 1-3 | 1 | 1 | 1-4 | 1 |
| Selfcare | before | 1 | 0 | 1-2 | 1 | 0 | 1-3 | 1 |
|  | after | 1 | 0 | 1-2 | 1 | 1 | 1-4 | 1 |
| Activity | before | 2 | 1 | 1-4 | 3 | 1 | 1-4 | 1 |
|  | after | 2 | 1 | 1-3 | 2 | 1 | 1-4 | 1 |
| Pain | before | 4 | 1 | 2-5 | 3 | 1 | 2-4 | 1 |
|  | after | 2 | 1 | 1-3 | 3 | 1 | 1-4 | 1 |
| Anxiety | before | 2 | 1 | 1-4 | 2 | 1 | 1-4 | 1 |
|  | after | 2 | 1 | 1-3 | 2 | 1 | 1-4 | 1 |
| EQ-5D-5L Index | before | 0.53 | 0.22 | 0.27-0.91 | 0.64 | 0.18 | 0.19-0.91 | 1 |
|  | after | 0.85 | 0.07 | 0.75-1.0 | 0.74 | 0.26 | 0.08-1.0 | 1 |
| Missed days at work | before | 7 | 5 | 3-16 | 14 | 17 | 0-72 | 1 |
|  | after | 5 | 4 | 0-11 | 8 | 12 | 0-54 | 1 |
| Performance at work < 50% | before | 15 | 13 | 4-50 | 27 | 23 | 0-80 | 1 |
|  | after | 9 | 6 | 0-20 | 16 | 19 | 1-60 | 1 |
| Inability to do household work | before | 15 | 9 | 3-29 | 21 | 19 | 2-72 | 1 |
|  | after | 9 | 5 | 3-15 | 14 | 16 | 0-60 | 1 |
| Household < 50% | before | 17 | 13 | 4-50 | 18 | 15 | 1-50 | 1 |
|  | after | 9 | 7 | 0-20 | 12 | 14 | 0-56 | 1 |
| Days missed social events | before | 14 | 13 | 4-45 | 18 | 20 | 0-80 | 1 |
|  | after | 7 | 6 | 0-20 | 11 | 15 | 0-54 | 1 |
| MIDAS Score | before | 69 | 36 | 27-140 | 98 | 75 | 6-243 | 1 |
|  | after | 38 | 18 | 14-67 | 61 | 63 | 5-235 | 1 |
| Headache days (last 3 months) | before | 35 | 27 | 3-91 | 49 | 26 | 3-92 | 1 |
|  | after | 24 | 14 | 7-63 | 35 | 28 | 2-90 | 1 |
| Headache intensity | before | 7 | 2 | 4-9 | 7 | 2 | 4-10 | 1 |
|  | after | 7 | 2 | 3-9 | 6 | 2 | 3-9 | 1 |
| MHD | before | 14 | 8 | 5-31 | 17 | 9 | 3-31 | 1 |
|  | after | 8 | 4 | 2-18 | 11 | 9 | 1-30 | 1 |
| MMD | before | 11 | 7 | 5-26 | 15 | 9 | 2-31 | 1 |
|  | after | 6 | 3 | 2-13 | 9 | 8 | 1-26 | 1 |
| MMD | before | 13 | 12 | 3-31 | 11 | 9 | 1-31 | 1 |
|  | after | 5 | 3 | 1-13 | 8 | 8 | 0-31 | 1 |

*MIDAS* Migraine Disability Assessment, *MHD* monthly headache days, *MMD* monthly migraine days, *MDD* monthly analgesic drug days.

**Supplemental Table 5:** **Medication overuse headache (MOH) and outcome parameters before and after addition of Erenumab.** Values before and after initiation of dual therapy were compared for patients with and without MOH via Wilcoxon matched-pairs signed rank test. An adjusted p-value of < 0.05 was considered statistically significant after Bonferroni correction.

|  |  | **No MOH (n=26)** | | | | **MOH (n=9)** | | | |
| --- | --- | --- | --- | --- | --- | --- | --- | --- | --- |
| **Variables** |  | Mean | SD | range | p-value | Mean | SD | range | p-value |
| Mobility | before | 2 | 1 | 1-4 | 1 | 2 | 1 | 1-3 | 1 |
|  | after | 2 | 1 | 1-4 |  | 1 | 1 | 1-3 |  |
| Selfcare | before | 1 | 0 | 1-3 | 1 | 1 | 1 | 1-2 | 1 |
|  | after | 1 | 1 | 1-4 |  | 1 | 0 | 1-1 |  |
| Activity | before | 2 | 1 | 1-4 | 1 | 3 | 1 | 1-4 | 1 |
|  | after | 2 | 1 | 1-4 |  | 2 | 1 | 1-3 |  |
| Pain | before | 3 | 1 | 2-5 | **0.017** | 3 | 1 | 3-4 | 0.5304 |
|  | after | 3 | 1 | 1-4 |  | 2 | 1 | 1-3 |  |
| Anxiety | before | 2 | 1 | 1-4 | 1 | 3 | 1 | 1-4 | 1 |
|  | after | 2 | 1 | 1-4 |  | 2 | 1 | 1-3 |  |
| EQ-5D-5L Index | before | 0.61 | 0.20 | 0.19-0.91 | 0.0697 | 0.59 | 0.18 | 0.29-0.8 | 0.0663 |
|  | after | 0.75 | 0.25 | 0.08-1.0 |  | 0.85 | 0.09 | 0.72-1.0 |  |
| Missed days at work | before | 11 | 16 | 0-72 | **0.0119** | 14 | 9 | 3-33 | 0.1326 |
|  | after | 6 | 11 | 0-54 |  | 10 | 8 | 3-26 |  |
| Performance at work < 50% | before | 22 | 21 | 0-80 | **0.0408** | 28 | 21 | 7-62 | 1 |
|  | after | 14 | 16 | 0-58 |  | 14 | 18 | 1-60 |  |
| Inability to do household work | before | 16 | 13 | 2-50 | 0.4131 | 29 | 23 | 3-72 | 1 |
|  | after | 11 | 12 | 0-54 |  | 16 | 19 | 1-60 |  |
| Performance household < 50% | before | 16 | 15 | 1-50 | 0.1037 | 23 | 11 | 11-40 | 0.6647 |
|  | after | 9 | 11 | 0-56 |  | 15 | 14 | 2-46 |  |
| Days missed social events | before | 15 | 18 | 0-80 | 0.2805 | 22 | 18 | 5-46 | 1 |
|  | after | 9 | 11 | 0-54 |  | 11 | 16 | 0-50 |  |
| MIDAS | before | 79 | 63 | 6-243 | **0.0068** | 117 | 72 | 39-224 | 0.9299 |
|  | after | 49 | 48 | 5-202 |  | 65 | 69 | 10-235 |  |
| Headache days (last 3 months) | before | 40 | 27 | 3-92 | **0.0391** | 55 | 26 | 30-92 | 1 |
|  | after | 30 | 25 | 2-90 |  | 37 | 27 | 7-80 |  |
| Headache intensity (VAS) | before | 7 | 2 | 4-10 | 0.5066 | 7 | 2 | 4-9 | 1 |
|  | after | 6 | 2 | 3-9 |  | 6 | 2 | 3-9 |  |
| MHD | before | 15 | 9 | 3-31 | **0.0017** | 20 | 9 | 10-31 | 0.1989 |
|  | after | 10 | 8 | 2-30 |  | 11 | 8 | 1-27 |  |
| MMD | before | 13 | 8 | 2-31 | **0.0017** | 17 | 8 | 5-31 | 0.1989 |
|  | after | 8 | 7 | 1-26 |  | 8 | 5 | 1-17 |  |
| MDD | before | 6 | 3 | 1-14 | 1 | 27 | 6 | 18-31 | 0.1326 |
|  | after | 6 | 6 | 1-27 |  | 9 | 9 | 0-31 |  |

*MOH* medication overuse headache *MIDAS* Migraine Disability Assessment, *VAS* visual analog scale, *MHD* monthly headache days, *MMD* monthly migraine days, *MDD* monthly analgesic drug days.

**Supplemental Table 6:** **Resistant migraine and outcome parameters before and after Erenumab addition.** Values before and after initiation of dual therapy were compared for patients with and without resistant migraine via Wilcoxon matched-pairs signed rank test. A p-value of <0.05 was considered statistically significant after Bonferroni correction.

|  |  | **No Resistant migraine (n=11)** | | | | **Resistant migraine (n=24)** | | | |
| --- | --- | --- | --- | --- | --- | --- | --- | --- | --- |
| **Variables** |  | Mean | SD | range | p-value | Mean | SD | range | p-value |
| Mobility | before | 2 | 1 | 1-4 | 1 | 2 | 1 | 1-4 | 1 |
|  | after | 1 | 1 | 1-3 |  | 1 | 1 | 1-4 |  |
| Selfcare | before | 1 | 0 | 1-2 | 1 | 1 | 0 | 1-3 | 1 |
|  | after | 1 | 0 | 1-2 |  | 1 | 1 | 1-4 |  |
| Activity | before | 2 | 1 | 1-4 | 1 | 3 | 1 | 1-4 | 0.2992 |
|  | after | 2 | 1 | 1-3 |  | 2 | 1 | 1-4 |  |
| Pain | before | 4 | 1 | 2-5 | 0.2329 | 3 | 1 | 2-4 | **0.0408** |
|  | after | 2 | 1 | 1-3 |  | 3 | 1 | 1-4 |  |
| Anxiety | before | 2 | 1 | 1-4 | 0.2652 | 2 | 1 | 1-4 | 1 |
|  | after | 2 | 1 | 1-3 |  | 2 | 1 | 1-4 |  |
| EQ-5D-5L Index | before | 0.53 | 0.22 | 0.27-0.91 | 0.0833 | 0.64 | 0.18 | 0.19-0.91 | 0.1105 |
|  | after | 0.85 | 0.07 | 0.75-1.0 |  | 0.74 | 0.26 | 0.08-1.0 |  |
| Missed days at work | before | 7 | 5 | 3-16 | 0.9962 | 14 | 17 | 0-72 | **0.0017** |
|  | after | 5 | 4 | 0-11 |  | 8 | 12 | 0-54 |  |
| Performance at work < 50% | before | 15 | 13 | 4-50 | 0.5984 | 27 | 23 | 0-80 | 0.1853 |
|  | after | 9 | 6 | 0-20 |  | 16 | 19 | 1-60 |  |
| Inability to do household work | before | 15 | 9 | 3-29 | 0.4641 | 21 | 19 | 2-72 | 0.6375 |
|  | after | 9 | 5 | 3-15 |  | 14 | 16 | 0-60 |  |
| Performance household < 50% | before | 17 | 13 | 4-50 | 0.5474 | 18 | 15 | 1-50 | 0.2329 |
|  | after | 9 | 7 | 0-20 |  | 12 | 14 | 0-56 |  |
| Days missed social events | before | 14 | 13 | 4-45 | 0.4318 | 18 | 20 | 0-80 | 0.4947 |
|  | after | 7 | 6 | 0-20 |  | 11 | 15 | 0-54 |  |
| MIDAS Score | before | 69 | 36 | 27-140 | 0.1666 | 98 | 75 | 6-243 | **0.0306** |
|  | after | 38 | 18 | 14-67 |  | 61 | 63 | 5-235 |  |
| Headache days (last 3 months) | before | 35 | 27 | 3-91 | 1 | 49 | 26 | 3-92 | **0.0153** |
|  | after | 24 | 14 | 7-63 |  | 35 | 28 | 2-90 |  |
| Headache intensity | before | 7 | 2 | 4-9 | 1 | 7 | 2 | 4-10 | **0.0017** |
|  | after | 7 | 2 | 3-9 |  | 6 | 2 | 3-9 |  |
| MHD | before | 14 | 8 | 5-31 | **0.0493** | 17 | 9 | 3-31 | **0.0017** |
|  | after | 8 | 4 | 2-18 |  | 11 | 9 | 1-30 |  |
| MMD | before | 11 | 7 | 5-26 | 0.1666 | 15 | 9 | 2-31 | **0.0017** |
|  | after | 6 | 3 | 2-13 |  | 9 | 8 | 1-26 |  |
| MDD | before | 13 | 12 | 3-31 | 0.1666 | 11 | 9 | 1-31 | 1 |
|  | after | 5 | 3 | 1-13 |  | 8 | 8 | 0-31 |  |

*MIDAS* Migraine Disability Assessment, *VAS* visual analog scale, *MHD* monthly headache days, *MMD* monthly migraine days, *MDD* monthly analgesic drug days.

**Supplemental Table 7:** **EQ-5D-5L and MIDAS Score results.** Metrics of the dual therapy group at a visit before baseline and at baseline were compared via Wilcoxon matched-pairs signed rank test. Bonferroni correction for multiple testing was performed and adjusted p-value < 0.05 was considered statistically significant.

|  | **Pre-Baseline (n=35)** | **Baseline dual therapy group (n=35)** | **Adjusted p-value** |
| --- | --- | --- | --- |
| **EQ-5D-5L** |  |  |  |
| Mobility | 1.63 ± 0.88 (1-4) | 1.66 ± 0.94 (1-4) | 1 |
| Selfcare | 1.14 ± 0.49 (1-3) | 1.20 ± 0.47 (1-3) | 1 |
| Activity | 2.31 ± 1.02 (1-4) | 2.46 ± 0.92 (1-4) | 1 |
| Pain | 3.46 ± 0.92 (1-5) | 3.46 ± 0.70 (2-5) | 1 |
| Anxiety | 2.49 ± 0.89 (1-4) | 2.29 ± 0.93 (1-4) | 1 |
| EQ-5D-5L index value | 0.63 ± 0.23 (0.1-0.9) | 0.61 ± 0.20 (0.19-0.91) | 1 |
| **MIDAS Score** | 87.11 ± 60.43 (5-249) | 89.06 ± 66.17 (6-243) | 1 |
| Missed days at work | 11 ± 15.25 (0-72) | 11.89 ± 14.47 (0-72) | 1 |
| Performance at work < 50% | 23.17 ± 21 (0-81) | 27.40 ± 23.80 (2-84) | 1 |
| Inability to do household work | 18.8 ± 13.93 (0-70) | 19.49 ± 16.61 (2-72) | 1 |
| Performance household < 50% | 19.37 ± 16.64 (0-64) | 17.80 ± 13.94 (1-50) | 1 |
| Days missed social events | 14.77 ± 16.20 (0-80) | 16.66 ± 17.71 (0-80) | 1 |
| **MHD, MMD and MDD** |  |  |  |
| Headache days (last 3 months) | 44.06 ± 27.77 (3-90) | 44.20 ± 27.14 (3-92) | 1 |
| Headache intensity (VAS) | 6.91 ± 1.54 (4-10) | 7.06 ± 1.68 (4-10) | 1 |
| MHD | 16.06 ± 8.80 (1-30) | 16.20 ± 8.96 (3-31) | 1 |
| MMD | 11.86 ± 8.37 (0-30) | 13.77 ± 8.20 (2-31) | 1 |
| MDD | 10.94 ± 8.41 (2-30) | 11.37 ± 9.81 (1-31) | 1 |

*Data are expressed as mean ± standard deviation (range).*

*OnaBoNT-A* onabotulinumtoxin A, *MIDAS* Migraine Disability Assessment, *MHD* monthly headache days, *MMD* monthly migraine days, *MDD* monthly analgesic drug days, *VAS* visual analog scale.

**Supplemental Figure**

**Supplemental figure 1: Changes in Monthly Headache Days (MHD), Monthly Migraine Days (MMD), Monthly Medication Days (MDD), and Headache Intensity (VAS) Before and After Treatment with OnaBoNT-A Monotherapy, Erenumab Monotherapy, and Dual Therapy.** This figure displays the violin plots showing the distribution of monthly headache days (MHD), monthly migraine days (MMD), monthly medication days (MDD), and headache intensity (VAS) before and after treatment with OnaBoNT-A monotherapy, erenumab monotherapy, and dual therapy (OnaBoNT-A plus erenumab). Panels A, B, and C: Monthly Headache Days (MHD): (A) OnaBoNT-A monotherapy: Significant reduction in MHD after treatment. (B) Erenumab monotherapy: No significant change in MHD after treatment. (C) Dual therapy: Significant reduction in MHD after treatment. Panels D, E, and F: Monthly Migraine Days (MMD): (D) OnaBoNT-A monotherapy: Significant reduction in MMD after treatment. (E) Erenumab monotherapy: No significant change in MMD after treatment. (F) Dual therapy: Significant reduction in MMD after treatment. Panels G, H, and I: Monthly Medication Days (MDD): (G) OnaBoNT-A monotherapy: No significant change in MDD after treatment (ns). (H) Erenumab monotherapy: No significant change in MDD after treatment. (I) Dual therapy: Significant reduction in MDD after treatment. Panels J, K, and L: Headache Intensity (VAS): (J) OnaBoNT-A monotherapy: No significant change in headache intensity after treatment (ns). (K) Erenumab monotherapy: No significant change in headache intensity after treatment. (L) Dual therapy: No significant change in headache intensity after treatment. Each violin plot shows the distribution of data points, with the width representing the density and the central red line indicating the median value. Statistical analysis was performed using the Kruskal-Wallis test with Dunn’s correction. The asterisk (*) denotes statistical significance with p < 0.05, while "ns" indicates no significant difference.
