# Supplementary figures and images for: Additional effect of erenumab for patients with chronic migraine treated with onabotulinumtoxin A—real-world data from a preliminary cohort study
Source: Front Neurol. 2024 Jun 26;15:1370503. doi: 10.3389/fneur.2024.1370503 (PMC11234259; doi:10.3389/fneur.2024.1370503)

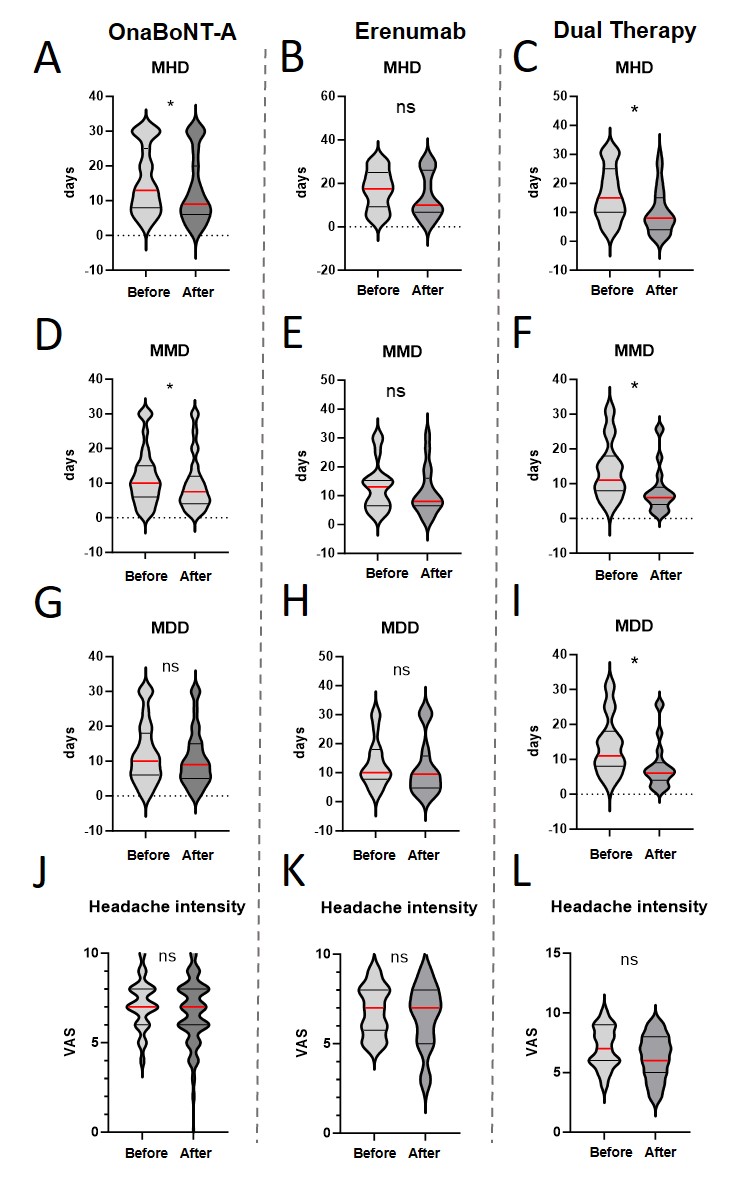

Supplement: Supplementary file 2 [file Image_1.JPEG]
